# Purifying Selection Bias against Microsatellites in Gene Rich Segmental Duplications in the Rice Genome

**DOI:** 10.1155/2012/970920

**Published:** 2012-09-13

**Authors:** P. C. Sharma, Manish Roorkiwal, Atul Grover

**Affiliations:** ^1^University School of Biotechnology, Guru Gobind Singh Indraprastha University, Sector 16C, Dwarka, New Delhi 110078, India; ^2^Centre of Excellence in Genomics, International Crops Research Institute for the Semi-Arid Tropics, Patancheru, Hyderabad 502324, India; ^3^Biotechnology Division, Defence Institute of Bio-Energy Research, Goraparao, Haldwani 263139, India

## Abstract

Little data is available on microsatellite dynamics in the duplicated regions of the rice genome, even though efforts have been made in the past to align genome sequences of its two sub-species. Based on the coordinates of duplicated sequences in the *indica* genome as available in the public domain, we identified microsatellites in these regions. CCG and GAAAA repeats occurred most frequently. In all, 259 microsatellites could be identified in the duplicated sequences using the criteria of minimum 90% alignability spread over a minimum of 1 Kb sequence. More than 25% of the repeats in duplicated regions occurred in the genic sequences. Only 45 (17%) of these 259 microsatellites were found conserved in the duplicated paralogues. Among these repeats, 40% maintained both sequence and length conservation. The effect of mutability of nearby regions could also be clearly seen in microsatellite regions. The overall purpose of this study was to investigate, whether microsatellites follow an independent course of evolutionary dynamics subsequent to events like genome reshuffling that simply drives these elements to different locations in the genome. To the best of our knowledge, this is the first comprehensive analysis of microsatellite conservation in the duplicated regions of any genome.

## 1. Introduction

 Microsatellites represent a class of tandem DNA repeats with 1 to 6 bp long repeat units. These sequences occur in almost all the organisms and frequently constitute the hypervariable regions of the genome. No specific functions have been assigned to most of the microsatellites till date. However, in some cases at least, microsatellite alleles provide protective or adaptive advantage to the host [1]. In many cases, occurrence of different alleles has been found associated with different phenotypes [2]. Microsatellites are not expected to be conserved for long evolutionary periods either, as argued by Buschiazzo and Gemmell [3]. Nevertheless, models of microsatellite mutational dynamics have been developed based on comparison of orthologous microsatellite loci in related taxa [4–7]. However, whether these models also describe microsatellites at paralogous loci created by segmental changes within a genome remains to be investigated. 

Availability of whole-genome sequences for rice (*Oryza sativa *L.) allows analysis of noncoding DNA also within the segmentally duplicated regions in addition to the gene order, tandemly arranged genes (TAGs) and gene functions. A collective look emerging from different reports on mapping of duplicated regions in rice genome [8–10] reflects that these studies primarily focused on the analysis of genes in these regions. The strategy commonly used involved making blocks of genes, and mapping them elsewhere in the genome. In a way, the noncoding DNA, particularly, the repetitive DNA has been ignored due to nonemployment of methods suitable for this kind of mapping. Nevertheless, to understand the complete mechanism of speciation and genome evolution, the characterization of conserved noncoding DNA is equally important [11]. No information, to date, is available on the fate of microsatellites in newly duplicated locations. Signatures of ancient duplications, in terms of sequence similarity of genes, and their genomic order on chromosomes in rice, are widely available, as mapped by Yu et al. [10]. Using the same information as a reference, we have attempted to outline the dynamics of microsatellite DNA within the segmentally duplicated regions of the rice genome to enlighten the patterns of conservation and divergence of these sequences. The overall objective of this study was to investigate whether there is any participation of microsatellites in genome reshuffling or they are simply being carried over. We were also interested to know if after duplication the paralogous microsatellites (we call as “microsatellite twins”) follow independent dynamics as both the sites are now different or similar dynamics as the neighbouring environment is still essentially the same. The latter point is important to understand whether microsatellite hypermutability is random or directional.

## 2. Methods

### 2.1. Sequence Resources

Whole-genome sequence of *Oryza sativa *subspecies *indica *was downloaded from http://rise.genomics.org.cn/rice/index2.jsp (BGI release 2003-08-01) in FASTA format. Based on the coordinates of duplicated sequences as provided by Yu et al. [10], the sequences of duplicated regions were retrieved from the whole-genome sequence in a text editor and were used as plain text files. The first set of sequences described by Yu et al. [10] has been referred here as group I sequences, and their paralogous duplicated sequences have been designated as group II sequences. These sequences were further split into 2.0 Mb bins for further analysis. 

### 2.2. Analysis of Duplicated Sequences

Repeatmasker (http://www.repeatmasker.org/) with WU-blast [12] search engine was used with default sensitivity and rice as “DNA source” for mining of microsatellite repeats, which were subsequently aligned using glocal algorithm [13] in Vista Genome Browser (http://pipeline.lbl.gov/cgi-bin/gateway2) [14] following the method described earlier by Roorkiwal et al. [7]. A simple sequence with repeat motif length of 1–6 bp spanning a minimal length of 20 bp was considered as a microsatellite. Genes were predicted using MolQuest ver. 1.6.2 (Softberry; http://www.molquest.com/). Following analysis of the aligned map, segmental duplications were identified by the criteria of similarity >90% and length ≥1 Kb [15] and analysed for microsatellites and coordinates of the predicted genes. 

### 2.3. Statistical Analysis

The data generated by mining of duplicated sequences and associated microsatellites were subjected to statistical analysis using *χ* ^2^ test and correlation test. The expected values were derived from the published reports [5, 7, 10]. 

## 3. Results and Discussion 

Microsatellites constitute nearly 1% of the eukaryotic genomes, though in some organisms like *Plasmodium *they may be overrepresented [16]. Their biological significance to the host genomes has been a topic of debate in recent years. Moreover, little knowledge is available about their mutational dynamics [17, 18], primarily derived from the limited genomewide studies in model organisms [4, 5, 7]. Comprehensive surveys on microsatellite conservation across the species and within duplicated sequences of the same genome are, therefore, required to expand our understanding regarding their genomic significance. In the following sections, we present some points emerging from our study justifying our opinion that at least in part such a conservation and maintenance of microsatellites in segmentally duplicated sequences are visible in the rice genome. 

### 3.1. Alignability of Duplicated Regions

Evidences exist for genome duplications in rice that occurred between 53 and 94 mya sometime prior to divergence of the cereal genomes [9, 10]. Further, a segmental duplication event between chromosomes 11 and 12 occurred around 5 mya is also well documented [19], in addition to numerous other individual gene duplications [1, 9]. In totality, the duplicated sequences in rice span 295 Mb, representing nearly two-third of the entire genome including 47% of the genic regions [10]. It is believed that duplication events are followed by several genomic changes including loss of gene functions, and in certain cases, loss of entire genes also [9].

Based on the data presented earlier by Yu et al. [10], we delimited total duplicated regions as 141 Mb of group I sequences and 154 Mb of group II sequences. However, the actual traceable duplicated segments meeting the criteria of >90% similarity and minimum of 1 Kb [15] length covered merely 3.8 Mb genome. The first and second groups of sequences spanned 1.89 and 1.90 Mb of the genome, respectively. Thus, the actual portion of the rice genome studied here came out to be merely 1% (~3.79 Mb). Maximum duplication events were observed on chromosome 2 (~0.34 Mb) and minimum on chromosome 7 spanning little lesser than 0.1 Mb (Table 1). Their distribution was obviously non random with *P*(*χ* ^2^) < 0.001. Further, no correlation was observed between the size of duplicated segments and the length of chromosomes. Average length of bins was found highest on chromosome 5, and minimum on chromosome 6. 

**Figure 1:**
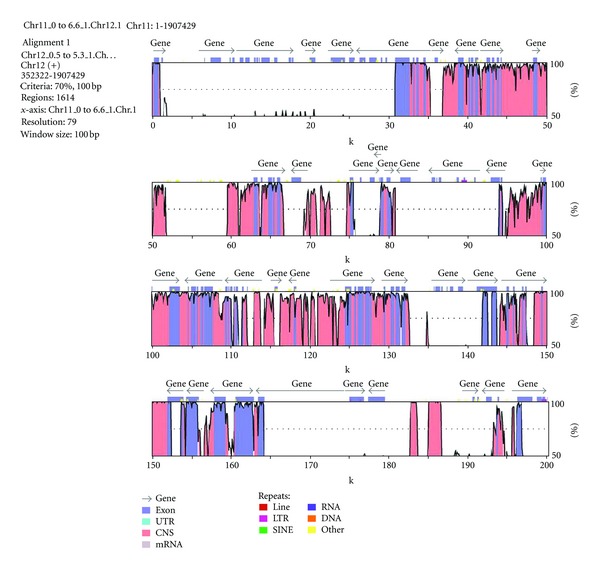
A representative figure of a duplicated segment mapped between chromosomes 11 and 12.

The size of the aligned pair and the alignment scores between two segments are generally in inverse relationship to their divergence time. However, in the present case, such a relationship has not been observed, as the most recent pair of duplicated sequences on chromosome 11 and 12 [19] was not the longest one (Table 1). Nevertheless, the mean similarity between the duplicated bins on chromosome 11 and 12 (Figure 1) was little higher at 94%, compared to mean similarity of 93.5% between duplicated bins of chromosome 2 and 4. 

### 3.2. Microsatellite Abundance in Duplicated Regions

**Figure 2:**
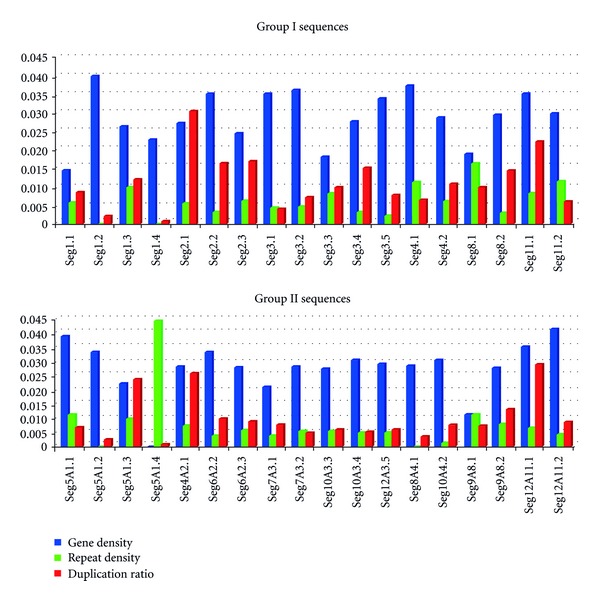
Gene versus repeat density on the entire duplicated segments in the rice genome. Duplication ratio refers to the ratio of the segment reported duplicated by Yu et al. [10], and the length of the fragments that we found aligning with >90% similarity for a minimum length of 1 Kb.

We earlier reported 45,782 microsatellites in 374.5 Mb of rice genome [7] using the same criteria and the tools used in the present study. Accordingly, 1% of the genome should have carried 458 microsatellites, had they been randomly distributed throughout the genome. However, only 259 microsatellites could be identified in this set of sequences, with 121 sequences identified in shorter set of 1.89 Mb, with an average frequency of one repeat locus per 16,453 bp, and 138 in group II of 1.9 Mb with average frequency of one repeat locus per 15,831 bp (Figure 2). When the frequency of specific microsatellite motifs in duplicated regions were plotted against the expected values, based on previous studies [5, 7], frequency of most of the microsatellites were found much lower *P*(*χ* ^2^) < 0.001, except for motifs like AAT, AGC, and CCG for which observed values corresponded to expected values. Clearly, there is certain level of purifying selection against the microsatellites in these duplicated regions of the rice genome.

**Figure 3:**
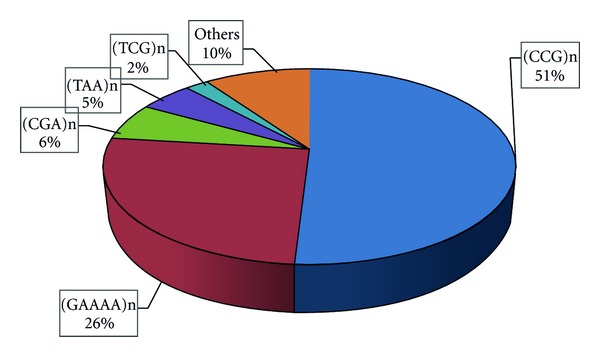
Abundance of microsatellite motifs in duplicated regions of the rice genome.

CCG repeats (and direct and reverse complementary permutations thereof) were found most abundant in either set of sequences in consistency with the earlier reports [5, 7]. GAAAA repeats (and their permutations), known to be most abundant in rice genome among the penta-nucleotide repeats [5], were found the second most abundant and least mutable repeats (Table 2) among the duplicated sequences. Other repeats like A, AT, and so forth, otherwise abundant in rice genome, were not found preferentially distributed in duplicated regions (Figure 3). Relative abundance of each of the repeat motif in both of the sets of sequences was fairly comparable. Quite expectedly, majority of the microsatellites occurred in the intergenic sequences (Table 2), and least in the exonic sequences. Consistent with the previous findings [7], CCG repeats most frequently occurred in exonic sequences. As suggested earlier by some researchers [17, 18], intrinsic factors specific to the host genome and microsatellite themselves like repeat length, repeat sequence, neighboring genomic sequences, and so forth, are responsible for differential occurrence and conservation of microsatellites. Importantly, while the duplicated sequences have shown a higher frequency of genes, they have particularly shown a bias against the microsatellites (Figure 2).

### 3.3. Microsatellite Conservation within the Duplicated Sequences

Out of the 259 microsatellites existing in the duplicated sequences, only 45 (17%) were found conserved in the paralogous sequences. Considering the mutability of microsatellites per locus per generation in rice, as described by Grover et al. [5], a microsatellite of 20 bp length may entirely be lost in around 2 million years provided all the mutations are unidirectional, targeting the shortening of the microsatellite. Thus, conservation of 17% of microsatellites in duplicated regions, with the average age of duplication around 56 mya, is especially significant as only 1% of the entire duplication blocks is identifiable today (discussed above). Interestingly, 42% of these repeats have their length conserved, which is significantly lesser than the global average in rice observed earlier [7], but clearly indicating that these alleles have been fixed in duplicated segments, most probably due to the vitality of their spatial occurrence [18]. Differences in the lengths of at least two paralogous microsatellites (with CCG motif) falling in exonic sequences on duplicated blocks on chromosome 11 and 12 indicate the relative advantage of repeatability and hypermutability of microsatellites in genes, as has been suggested earlier as well [1, 3, 20–22].

It was also interesting to note that at some of the genomic positions a single microsatellite repeat corresponded to two microsatellite repeats with the same motif (Table 3). This is possible due to recurring splitting and expansion events at microsatellite loci [18]. Of all the paralogous microsatellites observed, 40% maintained both sequence and length characteristics. Majority of these microsatellites were located on duplicated segments of chromosomes 1 and 5. It is quite possible, that these loci might have been fixed. However, we do not overrule the possibility that one or both of the sequences have undergone a number of mutations purely in stochastic manner and eventually arriving to the same lengths simultaneously, now seen as conserved alleles. Out of these two possibilities, it is the first one that generates more interest, as microsatellites associated with important regions in the genome will display lower variability during genetic drift and selective sweeps [18, 23]. Consequently, lesser activity will be observed on a microsatellite locus that is lying next to a genomic region adapted to a given environment [24]. Therefore, we do not overrule the possibility that the microsatellites that show sequence as well as length conservation represent important “evolutionary chronometers” [25] and might have been tightly linked to genomic regions of significance [18]. Microsatellites located in mutationally constrained regions are expected to be maintained passively. Highly conserved microsatellites are often associated with other conserved genomic elements and show a stronger negative relationship with single nucleotide polymorphisms (SNPs) density [26]. Interestingly enough, in five instances, a particular microsatellite motif has given way to another motif, precisely at the same site (Table 3). Grover and Sharma [18] explained such events by calling them as “metamorphosis” at microsatellite sites. Apparently, in three of the five cases, the new microsatellites appeared originally by a single site substitution, which later expanded possibly by “polymerase slippage” to mature into a fully grown microsatellite. Evidently, both the abundance and conservation of microsatellites had a heterogeneous pattern across the rice chromosomes. However, the distribution of sequence motifs across the chromosomes and across the blocks and segments of duplications more or less remained the same. Conserved microsatellites within the duplicated regions of the genome are desired candidates to study the overall significance of microsatellite conservation in different genomes. 

### 3.4. Microsatellites versus Genes in Segmentally Duplicated Regions

Out of 259, only 68 (26.25%) microsatellites were found to be associated with genes. Out of these genic microsatellites, 17 (25%) were present in exonic regions and remaining 51 (75%) were located in the intronic regions. Interestingly, 18 of the repeats and their counterparts were located to different genomic entities. For example, while one locus was located in the intergenic region, its paralgoue occurred in the genic region. Such spatial distribution can occur due to homologous recombination [27] or some other minor genomic rearrangements due to retrotransposition, local genomic reorganization and reshuffling. Thus, such microsatellites can be considered as “genomic fossils,” which can help in retracing the evolutionary events in the genome.

## Figures and Tables

**Table 1:** Occurrence of genes and microsatellite repeats in duplicated regions of the rice genome.

	Duplicated segments (length covered in bp)	Intergenic	Genic		Gene frequency (bp/gene)	Repeat frequency (bp/repeat)
			Exon	Intron		
Chromosome 1 corresponding chromosome 5						
Segment 1.1	58 (81685)	46	9	3	6807.08	16337
Segment 5A1.1	50 (75106)	21	20	9	2589.86	8345.11
Segment 1.2	6 (9866)	2	3	1	2466.5	0
Segment 5A1.2	4 (5909)	2	1	1	203.76	0.00
Segment 1.3	163 (244228)	98	42	23	3757.35	9769.12
Segment 5A1.3	169 (268072)	110	41	18	9243.86	9928.59
Segment 1.4	3 (4300)	2	0	1	4300	0
Segment 5A1.4	1 (2247)	1	0	0	77.48	2247.00

Chromosome 2 corresponding chromosomes 4 and 6						
Segment 2.1	342 (518707)	200	89	53	3652.87	17886.45
Segment 4A2.1	347 (522868)	199	97	51	18029.93	13071.70
Segment 2.2	102 (149764)	49	43	10	2825.74	29952.8
Segment 6A2.2	105 (146574)	56	35	14	5054.28	24429.00
Segment 2.3	81 (124845)	50	14	17	4027.26	15605.63
Segment 6A2.3	77 (114157)	45	22	10	3936.45	16308.14

Chromosome 3 corresponding chromosomes 7, 10, and 12						
Segment 3.1	29 (42425)	14	11	4	2828.33	21212.5
Segment 7A3.1	31 (47154)	21	9	1	1626.00	23577.00
Segment 3.2	29 (41410)	14	12	3	2760.67	20705
Segment 7A3.2	36 (49456)	22	9	5	1705.38	16485.33
Segment 3.3	37 (59771)	26	5	6	5433.73	11954.2
Segment 10A3.3	42 (66214)	24	9	9	2283.24	16553.50
Segment 3.4	23 (28749)	15	1	7	3593.63	28749
Segment 10A3.4	28 (39198)	16	6	6	1351.66	19599.00
Segment 3.5	29 (41024)	15	10	4	2930.29	41024
Segment 12A3.5	24 (37014)	13	8	3	1276.34	18507.00

Chromosome 4 corresponding chromosomes 8 and 10						
Segment 4.1	17 (26044)	7	6	4	2604.4	8681.33
Segment 8A4.1	16 (21129)	11	4	1	728.59	0.00
Segment 4.2	40 (62581)	22	12	6	3476.72	15645.25
Segment 10A4.2	40 (59065)	22	11	7	2036.72	59065.00

Chromosome 8 corresponding chromosome 9						
Segment 8.1	28 (36632)	21	4	3	5233.14	6105.33
Segment 9A8.1	33 (42741)	28	3	2	1473.83	8548.20
Segment 8.2	130 (191894)	73	47	10	3366.56	31982.33
Segment 9A8.2	122 (180824)	72	41	9	6235.31	12054.93

Chromosome 11 corresponding chromosome 12						
Segment 11.1	111 (168247)	51	40	20	2804.12	12017.64
Segment 12A11.1	101 (158798)	45	39	17	5475.79	14436.18
Segment 11.2	43 (59793)	25	15	3	3321.83	8541.86
Segment 12A11.2	47 (65442)	20	18	9	2256.62	21814.00

**Table 2:** Traceability of microsatellites originating from group I sequences into group II sequences.

Motif	Region	Length (bp) in group I sequences	Traceability in group II sequences		
			Equal	Short	Long
Chromosome 1 corresponding chromosome 5			9	2	2
(CCG)n	Intergenic	58	*√*		
(CCG)n	Intergenic	78		*√*	
(CGG)n	Intergenic	60			*√*
(CGG)n	Intergenic	60			*√*
(GAAAA)n	Intergenic	26	*√*		
(GAAAA)n	Intergenic	33		*√*	
(TTTTC)n	Intergenic	26	*√*		
(TTTTC)n	Intergenic	26	*√*		
(TTTTC)n	Intergenic	26	*√*		
(TTTTC)n	Intron	26	*√*		
(TTTTC)n	Intron	26	*√*		
(TTTTC)n	Intergenic	22	*√*		
(TTTTC)n	Intergenic	26	*√*		

Chromosome 2 corresponding chromosome 4			6	2	4
(CCG)n	Intron	174		*√*	
(CGA)n	Intron	150			*√*
(CGA)n	Intron	150			*√*
(CGG)n	Intergenic	58	*√*		
(CGG)n	Intergenic	58	*√*		
(CGG)n	Intergenic	211			*√*
(CGG)n	Intergenic	126			*√*
(CGG)n	Intergenic	211		*√*	
(GAAAA)n	Intergenic	28	*√*		
(TTTTC)n	Intron	22	*√*		
(TTTTC)n	Intergenic	28	*√*		
(TTTTC)n	Intergenic	27	*√*		

Chromosome 2 corresponding chromosome 6			2	1	2
(CCG)n	Intron	74			*√*
(CCG)n	Intergenic	123			*√*
(CCG)n	Intergenic	75		*√*	
(TTTTC)n	Intron	27	*√*		
(TTTTC)n	Intergenic	27	*√*		

Chromosome 3 corresponding chromosomes 7, 10, and 12			0	0	2
(CGG)n	Intergenic	59			*√*
(GAAAA)n	Intergenic	22			*√*

Chromosome 4 corresponding chromosomes 8 and 10			0	0	0

Chromosome 8 corresponding chromosome 9			1	2	1
(CCG)n	Intergenic	72			*√*
(CCG)n	Intergenic	155		*√*	
(CCG)n	Intergenic	199		*√*	
(TAA)n	Intergenic	29	*√*		

Chromosome 11 corresponding chromosome 12			1	2	1
(CCG)n	Exon	76			*√*
(CCG)n	Exon	154		*√*	
(CGG)n	Intergenic	147		*√*	
(TCG)n	Exon	70	*√*		

**Table 3:** Description of paralogous loci where microsatellite motif has been found altered either by splitting and integrating, or replaced with another motif.

Duplication pair	Motif at group I site	Motif at group II site
DP 1A5	(CCG)n	(TCC)n
	(TTAA)n	(CCG)n
	(CGG)n	(CCG)n
	(CGA)n	

DP 2A4	(CGG)n	(CGA)n

DP 8A9	(CCG)n	(CCG)n
	(TCG)n	
	(TAA)n	(CGA)n

DP 11A12	(CCG)n	(CCG)n
		(CCG)n
	(CGG)n	(CGA)n
	(CCG)n	(CCG)n
		(CCG)n
	(CCG)n	(CCG)n
		(CCG)n

## References

[1] Kashi Y, King DG (2006). Simple sequence repeats as advantageous mutators in evolution. *Trends in Genetics*.

[2] Wolf JBW, Harrod C, Brunner S, Salazar S, Trillmich F, Tautz D (2008). Tracing early stages of species differentiation: ecological, morphological and genetic divergence of Galápagos sea lion populations. *BMC Evolutionary Biology*.

[3] Buschiazzo E, Gemmell NJ (2010). Conservation of human microsatellites across 450 million years of evolution. *Genome Biology and Evolution*.

[4] Barbará T, Palma-Silva C, Paggi GM, Bered F, Fay MF, Lexer C (2007). Cross-species transfer of nuclear microsatellite markers: potential and limitations. *Molecular Ecology*.

[5] Grover A, Aishwarya V, Sharma PC (2007). Biased distribution of microsatellite motifs in the rice genome. *Molecular Genetics and Genomics*.

[6] Grover A, Ramesh B, Sharma PC (2009). Development of microsatellite markers in potato and their transferability in some members of Solanaceae. *Physiology and Molecular Biology of Plants*.

[7] Roorkiwal M, Grover A, Sharma PC (2009). Genome-wide analysis of conservation and divergence of microsatellites in rice. *Molecular Genetics and Genomics*.

[8] Guyot R, Keller B (2004). Ancestral genome duplication in rice. *Genome*.

[9] Wang X, Zhao X, Zhu J, Wu W (2005). Genome-wide investigation of intron length polymorphisms and their potential as molecular markers in rice (*Oryza sativa* L.). *DNA Research*.

[10] Yu J, Wang J, Lin W (2005). The genomes of *Oryza sativa*: a history of duplications. *PLoS Biology*.

[11] Retelska D, Beaudoing E, Notredame C, Jongeneel CV, Bucher P (2007). Vertebrate conserved non coding DNA regions have a high persistence length and a short persistence time. *BMC Genomics*.

[12] Altschul SF, Madden TL, Schäffer AA (1997). Gapped BLAST and PSI-BLAST: a new generation of protein database search programs. *Nucleic Acids Research*.

[13] Brudno M, Malde S, Poliakov A (2003). Glocal alignment: finding rearrangements during alignment. *Bioinformatics*.

[14] Frazer KA, Pachter L, Poliakov A, Rubin EM, Dubchak I (2004). VISTA: computational tools for comparative genomics. *Nucleic Acids Research*.

[15] Zhang L, Lu HHS, Chung WY, Yang J, Li WH (2005). Patterns of segmental duplication in the human genome. *Molecular Biology and Evolution*.

[16] Sharma PC, Grover A, Kahl G (2007). Mining microsatellites in eukaryotic genomes. *Trends in Biotechnology*.

[17] Bhargava A, Fuentes FF (2010). Mutational dynamics of microsatellites. *Molecular Biotechnology*.

[18] Grover A, Sharma PC (2011). Is spatial occurrence of microsatellites in the genome a determinant of their function and dynamics contributing to genome evolution?. *Current Science*.

[19] The Rice Chromosomes 11 and 12 Sequencing Consortia (2005). The sequence of rice chromosomes 11 and 12, rich in disease resistance genes and recent gene duplications. *BMC Biology*.

[20] Fondon JW, Garner HR (2004). Molecular origins of rapid and continuous morphological evolution. *Proceedings of the National Academy of Sciences of the United States of America*.

[21] Hancock JM, Simon M (2005). Simple sequence repeats in proteins and their significance for network evolution. *Gene*.

[22] Riley DE, Krieger JN (2009). UTR dinucleotide simple sequence repeat evolution exhibits recurring patterns including regulatory sequence motif replacements. *Gene*.

[23] Schlötterer C (2003). Hitchhiking mapping—functional genomics from the population genetics perspective. *Trends in Genetics*.

[24] Schlötterer C, Kauer M, Dieringer D (2004). Allele excess at neutrally evolving microsatellites and the implications for tests of neutrality. *Proceedings of the Royal Society B Biological Science*.

[25] Driscoll CA, Menotti-Raymond M, Nelson G, Goldstein D, O’Brien SJ (2002). Genomic microsatellites as evolutionary chronometers: a test in wild cats. *Genome Research*.

[26] Brandström M, Ellegren H (2008). Genome-wide analysis of microsatellite polymorphism in chicken circumventing the ascertainment bias. *Genome Research*.

[27] Brandström M, Bagshaw AT, Gemmell NJ, Ellegren H (2008). The relationship between microsatellite polymorphism and recombination hot spots in the human genome. *Molecular Biology and Evolution*.

